# Polysaccharide-based anticancer therapeutics: molecular mechanisms, translational advances, and the road toward precision oncology

**DOI:** 10.3389/fphar.2026.1757334

**Published:** 2026-04-20

**Authors:** Mulong Wei, Chenxia Gao, Shuai Li, Yuanyuan Wang, Jiatian Cao, Siqi Hao, Hongying Li, Huiming Zhang, Shuang Liu

**Affiliations:** 1 Medical College of Basic Sciences, Jiamusi University, Jiamusi, China; 2 Harbin Medical University, Harbin, China

**Keywords:** biological response modifiers, cancer therapy, drug delivery systems, immunomodulation, polysaccharides, tumor microenvironment

## Abstract

Polysaccharides comprise a structurally varied class of natural macromolecules found in plants, fungi, animals, marine algae, and microorganisms. Therefore, they have attracted considerable attention over several decades due to numerous anticancer-associated activities indicated by accumulating *in vitro* and *in vivo* evidence, along with clinical data of heterogeneous maturity—ranging from well-established adjuvants such as lentinan and PSK, which have demonstrated survival benefits in randomized controlled trials, to early-phase exploratory studies for agents like fucoidan. Traditional chemotherapeutic agents (e.g., alkylating agents, antimetabolites) exert direct cytotoxic effects; however, many contemporary small-molecule drugs—such as kinase inhibitors and hormone receptor modulators—act through targeted inhibition of oncogenic signaling, and immunotherapies (e.g., checkpoint inhibitors) function by enhancing endogenous anti tumor immunity rather than directly damaging tumor cells, as cytotoxic agents do. By contrast, polysaccharides are increasingly recognized as biological response modifiers that exert an impact on cancer development through, for instance, immune system functioning, redox, and/or inflammatory balance, communications between cancer and stromal cells of the tumor microenvironment, and intracellular signaling cascades. This review presents an outline of the structural variability, physiological sources, and functions of polysaccharides relevant to cancer treatment. Based on the present armamentarium of polysaccharides, the main modes of action are summarized in terms of immunomodulation through the engagement of pattern-recognition receptors, oxidative stress and inflammation regulation, quantifiable programmed cell death modes, angiogenesis and metastasis, and indirect regulation of oncogenic signaling pathways. These are first expressed in terms of the target-unrelated context and at the network level. Finally, we briefly discuss recent developments regarding polysaccharides as coadjuvants in chemotherapy, radiotherapy, and immunotherapy, in addition to their status as potential biomaterials in novel drug delivery systems. Critical reviews of relevant issues regarding structural heterogeneity and reproducibility, pharmacokinetics, and clinical translation are given. Indeed, this review presents polysaccharides as multi-functional components in multi-dimensional cancer therapy, paying due attention to appropriate structural elucidation, mechanism validation, and systems-oriented approaches to their rational development and clinical application.

## 1 Introduction

Cancer remains one of the leading causes of death globally and continues to represent a huge challenge for public health worldwide, despite great medical achievements such as surgical resection, chemotherapy, radiotherapy, targeted therapy, and immunotherapy. According to recent international cancer statistics, the annual incidence of most cancers is more than 19 million, with almost 10 million deaths, with high geographic heterogeneity in cancer incidence and mortality (Sung et al., 2021). Across many solid tumors, late-stage diagnosis, intratumoral heterogeneity, therapeutic resistance, and treatment-related systemic toxicity remain critical factors limiting durable clinical benefit. Consequently, the development of safe, effective, and multi-target anticancer agents that can complement existing therapeutic modalities remains an urgent priority in oncology research.

Natural product–derived therapeutics have long served as an important source of anticancer agents, among which polysaccharides have attracted increasing attention because of their broad biological activities and generally favorable safety profiles. Polysaccharides are naturally occurring macromolecules widely distributed in plants, fungi, animals, algae, and microorganisms. Accumulating evidence from cancer-related experimental studies indicates that certain bioactive polysaccharides exhibit anticancer-associated biological activities in specific contexts, including immunomodulation, regulation of oxidative stress and inflammatory responses, modulation of apoptosis- or autophagy-related processes, and interference with angiogenesis- and metastasis-associated events (Newman and Cragg, 2020; Turrini et al., 2023; Ying and Hao, 2023). Rather than acting as classical cytotoxic agents, polysaccharides are increasingly recognized as biological response modifiers that may influence tumor progression primarily through regulation of immune responses and intracellular signaling networks.

G. lucidum polysaccharides have already been reported in some representative studies as having anti-tumor effects through the activation of macrophages, dendritic cells, and cytokine-mediated immune responses in cancer-related experimental models (Ren et al., 2021) Other plant polysaccharides, such as Astragalus polysaccharide and red ginseng polysaccharide, have been implicated in tumor cell survival and immune regulation, depending on the experimental models. APS inhibiting breast cancer proliferation, migration, and invasion via EMT modulation, alongside broader effects like immune cell activation, cell cycle disruption, signal inhibition (PI3K/Akt, MAPK/ERK, NF-κB), autophagy activation, and TME modulation. (Yang et al., 2024; He et al., 2024). Polyporus umbellatus polysaccharides and other medicinal fungi polysaccharides have shown anti-tumor and immunostimulatory effects both *in vitro* and *in vivo* in some cancer models, and *in vivo* antitumor activity has been reported for related fungal polysaccharides in other tumor models (Li T. et al., 2025; Liang et al., 2021; Liu et al., 2020).

Except for the direct effect on tumor cells, polysaccharides have shown a regulatory effect on the essential elements of TME-immune cells polarization, cytokines secretion, and matrix interactions. This might explain the modulation of chemotherapy, radiotherapy, and immunotherapy observed in various preclinical cancer models (Wang W. et al., 2024; Ying and Hao, 2023; Deng et al., 2024). Indeed, the tumor extracellular matrix rich in chondroitin-6-sulfate has been implicated in stromal macrophage polarization and immune exclusion via the JAK/STAT3 and Hedgehog pathways. On the other hand, its effect is partially blocked by surfen, a small-molecule glycosaminoglycan-binding antagonist that can reduce immunosuppression (Wu et al., 2022; Schuksz et al., 2008). Most recently, the anti-tumor effect of the exopolysaccharide EPS364, isolated from a deep-sea bacterium, toward hepatocarcinoma cells and cell adhesion was found to be mainly related to downregulation of the FGF19–FGFR4 pathway (Wang Y. et al., 2021).

Compared with traditional small-molecule anticancer drugs, polysaccharides are mostly multi-target molecules with low systemic toxicity and good biocompatibility; therefore, they are used as adsorbents of anticancer drugs or drug delivery systems for targeted and controlled release, possessing very little cytotoxicity themselves (Wu et al., 2023; Yadav et al., 2022). Because of their abundant functional groups, polysaccharides can be chemically manipulated and incorporated into new drug delivery systems, such as nanoparticles, hydrogels, and conjugated carriers, increasing potential translational applications (Yadav et al., 2022; Kurczewska, 2022).

**FIGURE 1:**
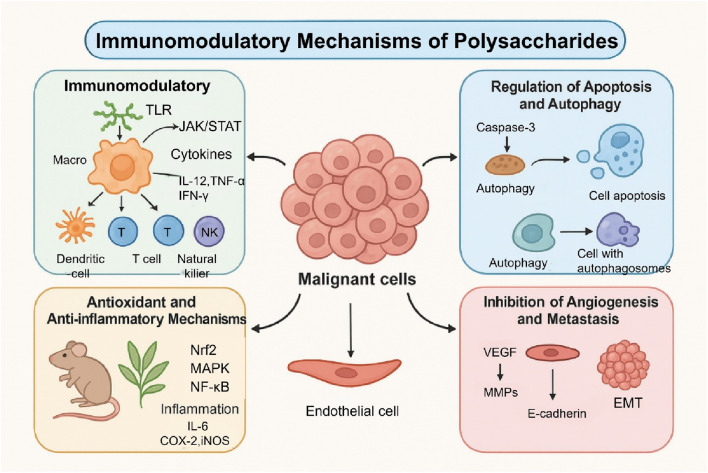
Overview of the anticancer-associated biological activities of natural polysaccharides. The diagram summarizes the complex modulation of biological signaling networks, including immunomodulation (TLR/JAK/STAT), induction of apoptosis (caspase-3) and autophagy, antioxidant/anti-inflammatory effects (Nrf2/NF-κB), and suppression of tumor angiogenesis and metastasis (VEGF/EMT). These diverse activities suggest that polysaccharides serve as potent adjuncts in cancer therapy by interfering with multiple cancer-related signaling events. Abbreviations: TLR, Toll-like receptor; NK, natural killer; VEGF, vascular endothelial growth factor; EMT, epithelial-mesenchymal transition; MMPs, matrix metalloproteinases.

Notwithstanding these good properties, many polysaccharide based systems remain difficult to translate clinically in cancer therapy; the source of polysaccharides, extraction and purification protocols, and intrinsic structural heterogeneity contribute to poor reproducibility and mechanistic definition, posing substantial challenges for quality control. Moreover, Natural polysaccharides are widely used as building blocks for advanced drug delivery systems, yet challenges in understanding their *in vivo* behavior and controlling drug release still limit fully optimized dosing and clinical translation (Liu Y. et al., 2025; Visan and Cristescu, 2023). Hence, this review summarizes polysaccharides’ bioactivities and their underpinning molecular mechanisms in cancer therapy, followed by a review of the latest advances in combination treatment strategies and polysaccharide DDSs. Finally, we summarize the consequent clinical research and translational problems. To explain the level-integrated mechanisms through which polysaccharides modulate tumor progression and therapy responses, an integrative conceptual framework of the most important bioregulatory actions of polysaccharides and interactions is schematically summarized in Figure 1.

## 2 Biological sources and structural characteristics of polysaccharides

Polysaccharides are large-size macromolecular organisms with carbohydrate monomers linked via glycosidic bonds, found in various biological resources, including plants, fungi, animals, marine macroalgae, and microorganisms. Structural variability exists due to the monosaccharide composition, glycosidic linkage type, branching pattern, molecular weight, and chemical substitution, exhibiting a wide range of biological activities. Further evidence from biochemical and cancer-related studies has indicated that such specific structural properties are reflected in polysaccharide binding to cellular receptors, signaling molecules, and tumor microenvironment components and that the functional consequence depends on the context in which this takes place (Visan and Cristescu, 2023; Liu Y et al., 2025).

Mechanistically, this three-dimensional conformation and chemical architecture define its recognition by various pattern-recognition receptors, interactions with other extracellular matrix components, and its mode of enzymatic cleavage. Based on this, structure-function relationships constitute a key concept for understanding the biological activities of polysaccharides and their rational design for therapeutic or biomaterial uses (Goodridge et al., 2011). In this review, polysaccharides are categorized according to biological origin into plant-derived, fungal-derived, animal-derived, marine algal–derived, and microorganism-derived polysaccharides. For clarity, microorganism-derived polysaccharides primarily refer to bacterial and archaeal polysaccharides, including extracellular and capsular polysaccharides (EPS/CPS). Marine algal polysaccharides are presented as a distinct category from fungal polysaccharides, given their unique sulfation patterns and physicochemical properties, which differ substantially from those of terrestrial fungal β-glucans. To facilitate comparison across different biological sources, representative polysaccharides, together with their principal structural features and reported biological activities, are summarized in Table 1.

**TABLE 1:** Representative polysaccharides from different biological sources and selected biological activities reported in cancer-related studies.

Biological source	Polysaccharide	Main source	Chemical structure	Key structural features	Reported biological effects (cancer-related)/Clinical status	References
Plant	Pectin	Fruit peels (apple, citrus)	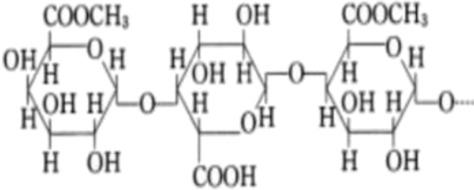	Acidic heteropolysaccharide; gel-forming	Reported to modulate gut microbiota, oxidative stress, and inflammatory responses in cancer-related models	Yue et al. (2023)
Plant	Astragalus membranaceus	*Astragalus membranaceus*	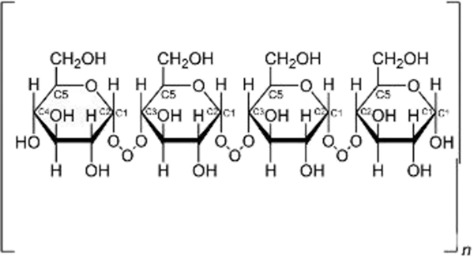	Branched heteropolysaccharides	Associated with immunomodulatory and supportive effects in tumor models. Investigational new drug (IND) stage in China; phase II trials for chemotherapy adjunct use ongoing	Tian et al. (2024)
Animal	Chondroitin sulfate	Cartilage and bone	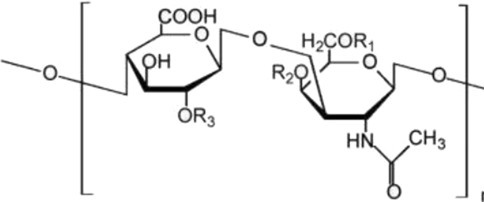	Sulfated glycosaminoglycan	Reported to influence tumor microenvironment and immune cell interactions. Primarily preclinical; chondroitin-6-sulfate isoform implicated in immune exclusion (Wu et al., 2022)	Yamada and Sugahara (2008)
Animal	Hyaluronic acid	Synovial fluid, connective tissue	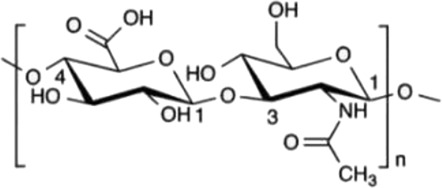	Linear anionic polysaccharide	Widely used as a biomaterial; associated with altered tumor–stroma interactions. FDA-approved as a pharmaceutical excipient; multiple hyaluronic acid-based nanomedicines in clinical trials for cancer therapy	Wu et al. (2024)
Fungal	Lentinan	*Lentinula edodes*	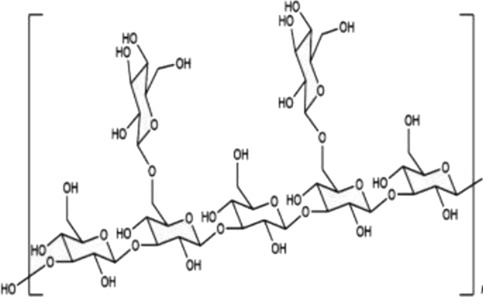	β-1,3-glucan with β-1,6 branches	Immunomodulatory activity; clinically approved as an adjuvant for gastric cancer in Japan since 1980s; also used for colorectal and lung cancer	Sheng et al. (2021)
Fungal	Ganoderma lucidum polysaccharides	*Ganoderma* spp.	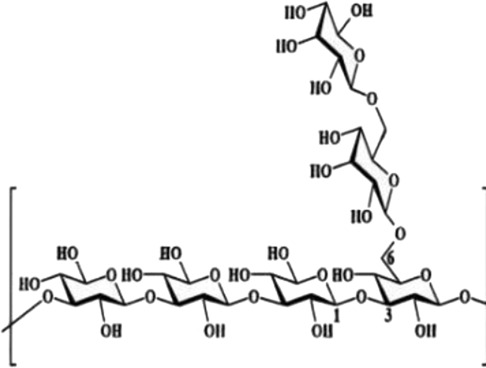	Heterogeneous polysaccharides; β-glucan fractions	Reported antioxidant and immunoregulatory effects in cancer-related studies. Extensively studied in preclinical and early-phase clinical settings; not approved as a standalone anticancer agent	Zhong et al. (2024)
Marine algae	Fucoidan	Brown algae	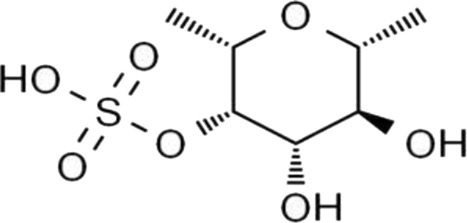	Sulfated fucan	Associated with immunomodulatory, anti-inflammatory, and anti-angiogenic effects in cancer models. Limited small-scale clinical trials as a dietary supplement; no regulatory approval for cancer indications	Turrini et al. (2023)
Microbial (EPS)	EPS364	Deep-sea bacteria	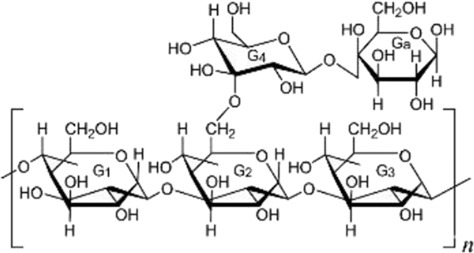	Exopolysaccharide	Reported to inhibit tumor cell growth and adhesion in preclinical liver cancer models. no clinical data available	Wang Y. et al. (2021)
Microbial (EPS)	Dextran	Lactic acid bacteria	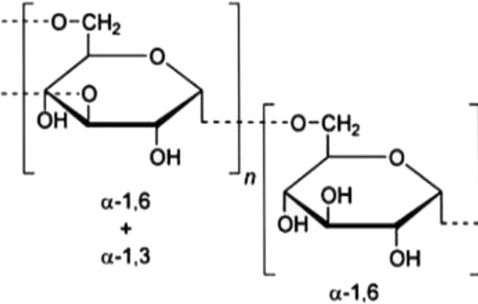	Branched α-glucan	Widely used as a drug carrier; explored in cancer drug delivery systems. FDA-approved pharmaceutical excipient; several dextran-based conjugates in preclinical and early-clinical development	Petrovici et al. (2023)

### 2.1 Major sources and classification of polysaccharides

Polysaccharides from different biological origins exhibit characteristic compositional and structural features that contribute to their functional diversity. Plant-derived polysaccharides, such as those isolated from Astragalus membranaceus, red ginseng (Panax ginseng), and Lycium barbarum, are typically heterogeneous polymers enriched in monosaccharides including arabinose, galactose, glucose, and rhamnose. These polysaccharides often contain mixed α- and β-glycosidic linkages and highly branched architectures, structural features that have been associated with immunomodulatory, antioxidant, and cytoprotective activities in cancer-related experimental models (Schepetkin and Quinn, 2006; Auyeung et al., 2016; Wang S. et al., 2021). In addition, pectin, an acidic heteropolysaccharide from fruit peels, has been shown to modulate gut microbiota and inflammatory responses in preclinical cancer models (Yue et al., 2023). Beyond direct effects on tumor cells, several plant-derived polysaccharides have been reported to enhance the efficacy of conventional chemotherapy and to alleviate treatment-associated toxicity in preclinical studies, supporting their potential use as adjunctive agents in cancer therapy (Wang S. et al., 2021; Zong et al., 2012).

Fungal polysaccharides have been one of the most studied classes of natural polysaccharides in oncological research. This is best illustrated by β-glucans from Ganoderma lucidum, Tremella fuciformis, Phellinus linteus, and Coriolus versicolor. Generally, such polysaccharides are often enriched in β-1,3 and β-1,6 glycosidic linkages and, in turn, are well known to exhibit macrophage-, dendritic-, and natural killer cell-activation effects and secretion of TNF-α, IL-12, and IFN-γ from these cells in different cancer models, as previously reviewed by Noorbakhsh Varnosfaderani et al. (2024); Ameri Shah Reza e al. (2026). Structurally, many fungal β-glucans have been reported to adopt triple-helix or coiled conformations, which are associated with recognition by pattern-recognition receptors such as Dectin-1 and complement receptor 3, thereby contributing to immune activation (Wang S. et al., 2021). It should be noted, however, that polysaccharide preparations derived from fungi are often heterogeneous; for example, Ganoderma polysaccharides frequently consist of mixtures of β-glucans, α-glucans, and heteropolysaccharides, with triple-helix conformations primarily described for selected purified β-1,3-glucan fractions (Boh, 2013).

Animal-derived polysaccharides, including chondroitin sulfate, hyaluronic acid, and heparin, are typically acidic macromolecules containing sulfate or carboxyl groups. These structural features enable strong interactions with extracellular matrix components and cell-surface receptors, supporting roles in cell adhesion, migration, and tissue remodeling (Cheung et al., 2025; Li M. et al., 2023; Gupta et al., 2024; Mikami and Kitagawa, 2023). In cancer-related contexts, animal-derived polysaccharides have been reported to modulate components of the tumor microenvironment, influence immune cell polarization, and serve as functional biomaterials for drug delivery applications. However, their biological effects are highly context dependent and are strongly influenced by molecular weight, sulfation degree, and structural heterogeneity (Afratis et al., 2012; Volpi and Linhardt, 2010; Pudełko et al., 2019).

Marine algal polysaccharides, primarily isolated from brown, red, and green seaweeds, include fucoidan, alginate, and carrageenan. These polymers are frequently characterized by a high degree of sulfation and strong negative charge density, which contribute to their interactions with growth factors, adhesion molecules, and angiogenesis-related mediators (Jiao et al., 2011). Fucoidan has been extensively investigated in cancer-related experimental models and has been reported to exhibit anti-angiogenic, anti-metastatic, and immunomodulatory activities. In contrast, alginate and carrageenan are more commonly explored as biomaterials for drug delivery and tissue engineering, although bioactive derivatives and modified forms have also been described.

Microorganism-derived polysaccharides primarily refer to bacterial and archaeal polysaccharides, including cell wall–associated polymers as well as extracellular and capsular polysaccharides (EPS/CPS). These polysaccharides exhibit substantial structural diversity and can be produced through controlled fermentation processes, offering advantages in scalability, batch-to-batch consistency, and reproducibility. Certain microbial polysaccharides have been reported to display anticancer-associated biological activities in preclinical models. For example, the deep-sea bacterium–derived exopolysaccharide EPS364 has been shown to inhibit liver cancer cell growth and adhesion in experimental systems, an effect associated with modulation of extracellular interactions and FGF19–FGFR4–related signaling pathways (Wang Y. et al., 2021). Owing to their tunable structures and amenability to chemical modification, microbial polysaccharides are increasingly explored as versatile platforms for anticancer drug delivery and biomaterial applications.

All in all, polysaccharides arising from different biological origins evidentially display network diversity, ranging from chemical composition and physicochemical properties to biological activities, inciting further evidence for meticulous structural elucidation in relation to biological activities’ interpretation and prudent extrapolation to the different eventualities of experimentation.

### 2.2 Structural features and structure–activity relationships

The bioactivities associated with the antitumor properties of polysaccharides have been related to the structural characteristics of the polysaccharides, such as monosaccharide composition, glycosidic linkage, branching, molecular weight, spatial conformation, and chemical modification. However, in recent years, bioactivities have been recognized not only as a function of an independent structural factor but rather as the combined effects of multivalent structures-multitudes of structures that contribute to receptor interaction, signaling modulation, bioactivity, and bioavailability of polysaccharides, as argued by Mao et al. (2025); Ying and Hao (2023).

In this regard, β-1,3 and β-1,6 branched glucans have been considered one of the best compounds to produce immunomodulatory activity, mainly because they interact with some receptors from the innate immune system, such as Dectin-1 and Toll-like receptors. However, the type of glycosidic linkage does not entirely characterize the biological activity; the immunomodulatory, antioxidative, and anti-inflammatory activities of the structurally complicated α-glucans and heteropolysaccharides depend on their branching architecture and molecular environment (Wang M., 2024; Liu L. et al., 2025; Huo et al., 2025; Fu et al., 2023) The second contributing factor is molecular mass. Most high molecular weight polysaccharides probably show their biological effects at or near the surface of the cell by receptor clustering and activation of immune signaling, while lower molecular weight fractions usually show better solubility, tissue distribution, and bioavailability (Anaya et al., 2023; Liu et al., 2023).

Moreover, it is the chemical sensitivity of polysaccharides to enzymatic degradation that essentially determines the gastrointestinal fate, systemic exposure, and especially the interactions of polysaccharides with the gut microbiota. For example, most fungal and microbial β-glucans are weakly digested by the host’s digestive enzymes, thus relatively well absorbed via GALT and fermented by the gut microbiota into short-chain fatty acids and related metabolites, which exert indirect immunomodulatory effects (Jayachandran et al., 2018; Tan et al., 2014) Different mechanisms act on classical storage polysaccharides, such as starch and glycogen. These are readily hydrolyzed by digestive enzymes and, therefore, are not recognized as immune-active macromolecules but as energy sources.

The physicochemical properties and biological performance of polysaccharides can largely differ upon chemical modification, such as sulfation, carboxymethylation, and acetylation. Up to now, polysulfation has been recognized as an important modification that increases the affinity of polysaccharides for positively charged protein domains and cell membranes and is associated with enhanced antiangiogenic, anticoagulant, and anticancer activities in experimental models (Liu et al., 2023; Figueroa et al., 2022) On the other hand, spatial conformations imply that triple helices are generally easier to be recognized by the immune system and show better immunomodulation, while flexible and linear chains are preferable for chemical derivatization and drug delivery system fabrication (Ying and Hao, 2023) Therefore, such structure–activity relationships, among others, form a conceptual framework within which we can understand the polysaccharide-associated biological activities and advance the purposeful design of chemically modified derivatives and polysaccharide-based delivery systems.

### 2.3 Correlation between structural features and antitumor functions

Polysaccharides involve very diverse biological resources and structural properties, thus exhibiting a variety of anticancer-associated biological activities. Indeed, although some regularity has been found in that fungal polysaccharides show immunomodulatory properties and marine sulfated polysaccharides show an antiangiogenic tendency, such regularity reflects a trend rather than a taboos-like law. Thus, polysaccharides from different sources act on similar biological outputs, such as modulating immune response, regulating oxidative stress and inflammation, and interfering with cancer-related signaling pathways (Ying and Hao, 2023; Yang et al., 2025; Turrini et al., 2023).

More often, animal polysaccharides and polysaccharide-based biomaterials display effects ascribed to structural determinants rather than differences in biological sources. For instance, through the organization of the extracellular matrix, the behavior of immune cells, and the distribution of drugs, the polymer modifies specific elements of the tumor microenvironment and thus may potentiate dissimilar therapeutic responses for applied experiments (Zhang et al., 2024; Zhou et al., 2025) These systemic effects align well with the perspective that polysaccharides are biological response modifiers capable of coordinating tumor/immune/stroma interactions rather than acting on a single molecular target (Ying and Hao, 2023; Li C. et al., 2025).

Polysaccharide structure elucidation is advancing in line with polysaccharide structure-function research, thanks to analytical techniques, high-field NMR, mass spectroscopy, and computational or bioinformatic modeling. These toolsets develop new understandings of the structure-function relationship further, making it possible to analyze detail of the monosaccharide component, its manner of linkage, the structure of branching, and higher-level conformations. From that perspective, polysaccharide research is in a transition from descriptive characterization to mechanistic and hypothesis-oriented research, which will rationally allow polysaccharides’ development as pharmaceuticals and their possible use in anticancer strategies (Wang B. et al., 2022).

## 3 Mechanisms of polysaccharides in cancer therapy

**FIGURE 2:**
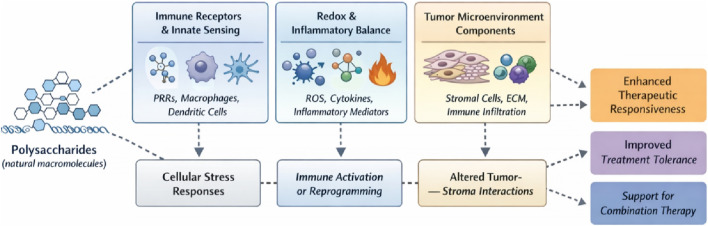
System-level framework illustrating biological effects associated with polysaccharide treatment in cancer-related models. System-wide, such relationships contribute to a better fit response to therapy and better tolerance of the treatments, above all when the anticancer standard therapies are given simultaneously. The relationships thus shown are context-dependent and primarily supported by preclinical data.

Polysaccharides evoke anticancer-related biological effects through a multilayered and tightly interrelated series of mechanisms. Unlike the chemotherapeutic small molecules that usually cause immense DNA damage or mitotic catastrophe during cell proliferation, polysaccharides are biological response modifiers that, through immune response modulation, redox, and inflammatory balances, apoptotic cell death pathways, angiogenesis, and intracellular signaling cascades, modulate the tumor progression process. Thus, most polysaccharides do not target a single molecular target but reconfigure the components of the tumor tissue microenvironment and deviate perturbed cellular signaling toward less dysregulated states. Following are their suggested molecular and cellular mechanisms, as reviewed by (Ahmad MF et al., 2024; Knelson et al., 2014; Ying and Hao, 2023). To provide an overview of the mechanisms pertaining to specific polysaccharide anticancer effects, a schematic review is provided in Figure 2.

### 3.1 Immunomodulatory mechanisms

Many antitumor polysaccharides, such as lentinan and yeast-derived β-glucans, are recognized as pathogen-associated molecular patterns (PAMPs) by pattern-recognition receptors (PRRs) on innate immune cells. Currently, immunomodulation stands out as one of the best-explored mechanisms by which polysaccharides exert their anticancer-related biological effects. Many bioactive polysaccharides are recognized by pattern-recognition receptors (PRRs) expressed on innate immune cells, including Toll-like receptors (TLRs), Dectin-1, the mannose receptor, and complement receptor 3. Engagement of these receptors has been reported to activate downstream signaling pathways such as nuclear factor κB (NF-κB), mitogen-activated protein kinase (MAPK), and Janus kinase/signal transducer and activator of transcription (JAK/STAT), leading to enhanced cytokine production, antigen presentation, and coordination of innate and adaptive immune responses (Brown and Gordon, 2005; Knelson et al., 2014).

In this respect, fungal β glucans such as lentinan and polysaccharides from Ganoderma lucidum are among the most extensively studied fungal pattern recognition receptor (PRR) ligands, particularly in relation to dectin 1-mediated innate immune responses. Specifically, lentinan, a β-1,3-glucan with β-1,6 branches, is clinically utilized as an adjuvant for gastric and lung cancers due to its potent immunomodulatory activity (Sheng et al., 2021). In experimental models, they activated macrophages and dendritic cells, induced the secretion ofIL-12, TNF-α, and IFN-γ, and enhanced CTL and NK activities (Ahmad et al., 2024; Habtemariam, 2020). Specifically, certain plant-derived polysaccharides have been shown to modulate immune signaling in defined experimental models. For instance, Astragalus polysaccharides have been reported to upregulate costimulatory molecules and major histocompatibility complex (MHC) expression in dendritic cells, which may contribute to a Th1-skewed immune response under specific conditions (Liu et al., 2011; Shao et al., 2006). Other marine polysaccharides, such as fucoidan, have been shown to influence the population of immune cells within the microenvironment of the tumor in a preclinical model by decreasing the population of immunosuppressive phenotype macrophages and Tregs in particular (Chen et al., 2020; Deng et al., 2022).

It is important to note that PRR-mediated recognition of polysaccharides typically involves multivalent and relatively low-affinity interactions that promote receptor clustering rather than classical high-affinity ligand–receptor binding. Consequently, immunomodulatory outcomes are strongly influenced by polysaccharide structure, molecular weight, and local biological context, underscoring the need for receptor-anchored and structure-informed mechanistic validation (Goodridge et al., 2011).

### 3.2 Regulation of apoptosis and autophagy

In addition to immunomodulation, polysaccharides have been reported to influence programmed cell death–related pathways, including apoptosis and autophagy, in a variety of cancer-related experimental models. Apoptosis-associated effects observed following polysaccharide treatment frequently involve modulation of mitochondrial signaling pathways, characterized by altered expression of pro-apoptotic and anti-apoptotic proteins, activation of caspase cascades, and disruption of mitochondrial membrane potential. Such effects have been described for polysaccharides derived from plant, fungal, and marine sources under specific experimental conditions (Wang Y. et al., 2024; Zhang et al., 2013).

The role of autophagy in cancer is characterized by a dual nature, functioning as either a survival mechanism or a contributor to cell death depending on the cellular context (White, 2015; Amaravadi et al., 2016). Correspondingly, the regulation of autophagy by polysaccharides appears to be highly context-dependent, varying according to tumor type, polysaccharide structure, and treatment conditions. In certain models, specific glycans such as β-glucans or Astragalus polysaccharides have been shown to induce autophagy-associated cell death or facilitate apoptosis (Zhu et al., 2023; Ying and Hao, 2023). These opposite effects highlight the dual role played by autophagy throughout cancer biology and warn against generalized interpretations of polysaccharide-induced autophagy without appropriate mechanistic substantiation.

In short, polysaccharides are generally not thought to act directly on intracellular apoptotic or autophagic machinery. Changes in the programmed death process induced by polysaccharides are therefore thought to be indirectly mediated downstream of cell surface receptor activation, immune-mediated signaling, or redox and metabolic cellular changes (Galluzzi et al., 2015; Moon et al., 2025).

### 3.3 Antioxidant and anti-inflammatory mechanisms

In this context, both oxidative stress and inflammation are important hallmarks of tumorigenesis and progression. In fact, the antioxidant and anti-inflammatory activities of most polysaccharides could be widely related to their biological effects associated with anticancer activity. These anticancer properties are frequently attributed to the targeted modulation of redox-sensitive and inflammatory signaling pathways. Specifically, certain polysaccharides have been shown to activate the nuclear factor erythroid 2–related factor 2 (Nrf2)/Kelch-like ECH-associated protein 1 (Keap1) axis, thereby enhancing antioxidant defenses and mitigating oxidative stress-induced DNA damage (Chen et al., 2024). Concurrently, these glycans can regulate mitogen-activated protein kinase (MAPK) and nuclear factor κB (NF-κB) signaling to suppress chronic inflammation-driven tumor progression (Cui and Shang, 2025).

Polysaccharides derived from Ganoderma lucidum, Astragalus membranaceus, and other medicinal sources have been reported to enhance endogenous antioxidant defense systems by upregulating enzymes such as superoxide dismutase, catalase, and glutathione peroxidase in cancer-related experimental models (Ahmad et al., 2024). Through these combined effects on oxidative stress and inflammatory responses, polysaccharides may help limit inflammation-driven tumor progression and reduce oxidative damage to the tumor microenvironment.

### 3.4 Inhibition of angiogenesis and metastasis

Angiogenesis and metastasis are essential processes underlying tumor growth and dissemination. A number of polysaccharides, particularly sulfated marine polysaccharides such as fucoidan, have been reported to interfere with angiogenesis- and metastasis-associated events in cancer-related experimental models. These effects have been associated with altered expression or activity of angiogenic and invasion-related factors, including vascular endothelial growth factor (VEGF), hypoxia-inducible factor-1α (HIF-1α), matrix metalloproteinases (MMPs), and cell adhesion molecules (van Weelden et al., 2019).

Polysaccharides have also been reported to influence epithelial–mesenchymal transition–associated markers and cytoskeletal organization, thereby modulating tumor cell migration and invasion in specific experimental contexts (Li et al., 2019; Yang et al., 2020; Liu H. et al., 2025). However, as observed for other biological mechanisms, anti-angiogenic and anti-metastatic effects are strongly dependent on polysaccharide structure, degree of sulfation, molecular weight, and experimental conditions. In many cases, inhibition of angiogenesis and metastasis likely reflects integrated effects on tumor cells, stromal components, and immune-mediated regulation within the tumor microenvironment rather than direct inhibition of individual angiogenic signaling pathways.

### 3.5 Modulation of oncogenic signaling pathways

Polysaccharides are increasingly recognized as broad modulators of intracellular signaling networks implicated in cancer progression. Rather than acting as direct inhibitors of oncogenic kinases or transcription factors, polysaccharides generally influence signaling pathways indirectly through upstream receptor engagement, immune-mediated signaling, and cytokine-dependent crosstalk. Multiple signaling axes have been reported to be affected in cancer-related experimental models, including phosphoinositide 3-kinase (PI3K)/Akt/mammalian target of rapamycin (mTOR), MAPK, NF-κB, Wnt/β-catenin, Janus kinase/signal transducer and activator of transcription (JAK/STAT), and Nrf2/Keap1 pathways as reviewed elsewhere (Ying and Hao, 2023; Mao et al., 2025).

Specifically, red ginseng polysaccharides have been shown to inhibit PI3K/Akt signaling and promote cell death in gastric cancer cells (Wang Y. et al., 2024), while other studies have explored the broader pharmacological networks of plant-derived compounds in cancer therapy (Li and Zhang, 2013). Specifically,a novel algae-derived polysaccharide has been shown to induce apoptosis and cell cycle arrest in certain cancer cell lines via activation of JNK signaling, independently of p38 MAPK. (Xie et al., 2018). NF-κB signaling represents another frequently implicated pathway, with polysaccharides reported to attenuate inflammatory gene expression and disrupt pro-survival transcriptional programs downstream of inflammatory stimuli (Habtemariam, 2020; Ying and Hao, 2023).

Some preclinical models report that polysaccharides indirectly regulate Wnt/β-catenin signaling, a key pathway in cancer stemness and metastasis, by modulating β-catenin stability and transcriptional activity (Yang et al., 2020; Liu H. et al., 2025). In addition, suppression of JAK/STAT signaling—particularly STAT3—has been associated with reduced immune evasion, pro-metastatic inflammation, and angiogenesis in polysaccharide-treated experimental models (Wei et al., 2024; Randeni and Xu, 2024).

### 3.6 Pathway crosstalk and network-level regulation

Polysaccharides can modulate several interconnected immune, inflammatory, and oncogenic signaling networks through defined receptors. This multi-pathway regulation may contribute to the broad effects observed in polysaccharide-based interventions. For instance, the activation of innate immune signaling may synergize with cytokine-dependent pathways to modulate downstream nodes such as the PI3K/Akt pathway, a concept supported by recent studies on polysaccharide-mediated immune reprogramming (Ying and Hao, 2023; Wang M. et al., 2024).

Polysaccharides often do not behave as classical mono-target drugs, which is consistent with the emerging paradigm of network pharmacology and systems biology. This complexity necessitates more rigorous receptor-based perturbation studies, pathway-based validations, and *in vivo* functional confirmations to support proposed mechanisms (Hopkins, 2008; Li and Zhang, 2013).

### 3.7 Translational implications of mechanistic complexity

All these mechanistic features have important implications for clinical translation. Their clinical relevance is best considered within an integrative view where immune modulation, redox balance, and inflammatory signaling networks are co-regulated, potentially enhancing the effectiveness or tolerability of classical anticancer therapies. This complexity reinforces the need for strict experimental design and biomarker-driven evaluation to validate specific receptors or pathways of interest (Hopkins, 2008).

**FIGURE 3:**
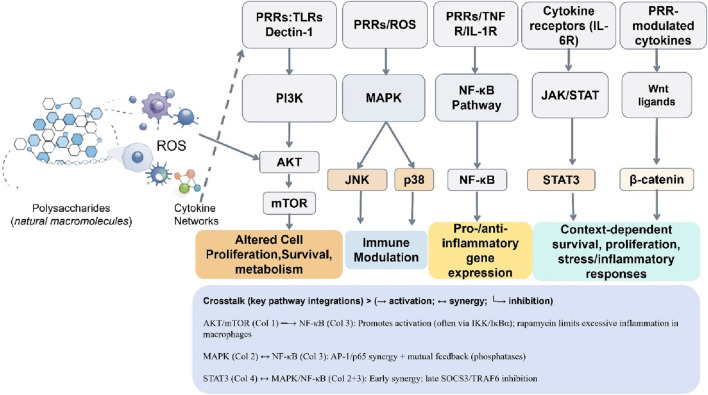
Schematic representation of the interconnected signaling networks modulated by polysaccharides. Left panel illustrates polysaccharides engaging immune cell PRRs, leading to ROS generation and cytokine production. A dashed feedback arrow from the cytokine network back to PRRs (Column 1) highlights cytokine-mediated modulation of receptor activity. Five parallel but interconnected columns depict major downstream pathways: (1) PI3K-AKT-mTOR, (2) MAPK, (3) NF-κB, (4) JAK-STAT, and (5) Wnt/β-catenin. Each pathway is linked to distinct yet overlapping outcomes in cell survival, metabolism, and immune regulation. A compact crosstalk box below summarizes key pathway integrations (e.g., AKT/mTOR, MAPK, and STAT3 mediated feedback) governing immune balance (→ activation; ↔ synergy; └→ inhibition).

Hence, polysaccharides are multipotent biological response modifiers, not direct cytotoxic entities-a position echoed by much of the evidence today. Further development in this field depends mainly on structural characterization, mechanism studies based on receptor engagement, and system integration analysis of biological functions into clinical therapy development pathways for new medicines (Ahmad et al., 2024; Tzianabos, 2000; Liu W. et al., 2025). Thus, in an attempt to integrate such diversified molecular, cellular, and microenvironmental effects into one broad view, a system-level network framework is proposed that may illustrate the polysaccharide anticancer activity in Figure 3.

## 4 Therapeutic applications and translational prospects of polysaccharides in cancer therapy

Polysaccharides are recognized as important immunomodulatory agents in modern cancer treatment, serving as valuable adjuvants to enhance the efficacy of classical therapies. The excitement surrounding polysaccharides stems from their good biocompatibility, structural versatility, roles as immune and signaling pathway modulators, and their promising potential as adjuncts, co-administered agents in combination regimens, and delivery matrices. However, their efficacy as monotherapies appears limited based on current evidence. Hence, the following paragraphs provide a summary of polysaccharide-based combination strategies, drug-delivery systems, and the onward pathway in clinical development for these agents, with a view to discussing translational opportunities and contemporary concerns.

### 4.1 Polysaccharides in combination therapy

One of the most rational translational approaches of polysaccharides could be combinations with standard anticancer treatments, including chemotherapy, radiotherapy, and immunotherapy. Indeed, robust evidence exists, supplemented by preclinical studies and reports from select clinical trials, that polysaccharides influence the responsiveness of therapy and relieve treatment-related adverse reactions by regulating immune function, redox balance, and inflammatory signals (Zhu et al., 2021; Ahmad et al., 2024; Wang D. et al., 2022; Chen et al., 2025).

Herbal polysaccharides, such as those derived from Astragalus, improved sensitivity to chemotherapeutic agents, such as cisplatin and 5-fluorouracil, in some experimental models. Mechanistically, this has been linked to the modulation of survival-related signaling pathways and the prevention of chemotherapy-induced immunosuppression rather than any obvious enhancement of cytotoxic synergy (Li et al., 2020; Wang et al., 2016). In general, fungal polysaccharides, such as lentinan and PSK, are used adjunctively in clinical practice; their primary function during cytotoxic therapies is to improve immune capacity, tolerance to treatment, and quality of life rather than to inhibit tumor growth directly (Habtemariam, 2020).

From this perspective, these polysaccharides have recently attracted much more attention in cancer immunotherapy. Some polysaccharides, in particular sulfated marine polysaccharides such as fucoidans, significantly elevate the antitumor immune response in immune checkpoint blockade preclinical models through the activation of the innate immunity system, cytokine production, and the infiltration of immune cells within the TME (van Weelden et al., 2019; Deng et al., 2026; Yang et al., 2021). It must be underscored that such observations are context-dependent and representative of experimental models; polysaccharides may be considered agents conditioning the immune system to increase response to immunotherapy, not as substitutes for immune checkpoint inhibitors.

Beyond their roles in chemotherapy and immunotherapy, polysaccharides have also been explored as potential adjuncts in radiation therapy due to their radioprotective and immunomodulatory properties. Recent studies suggest that certain polysaccharides may enhance antioxidant defenses and mitigate inflammation in healthy tissues, potentially improving treatment tolerability (Zhang et al., 2023; Ruiqing et al., 2025). These findings highlight the potential of polysaccharides as synergistic agents, though further validation in well-controlled clinical trials is necessary.

### 4.2 Polysaccharides as drug delivery systems and biomaterial platforms

Because of their inherent biological activities, polysaccharides also have potential as promising biomaterials for designing drug delivery systems due to their biodegradability, hydrophilicity, relative low immunogenicity, and abundant abundant functional groups that enable versatile chemical modifications. In fact, nanoparticles, micelles, hydrogels, and hybrid systems based on polysaccharides with polymers and metals have been developed in recent years to enhance solubility, stability, and tumor targeting capability of drugs (Banerjee et al., 2023).

Chemotherapeutic drugs loaded with carriers have also been extensively studied, including chitosan, hyaluronic acid, dextran, and alginate. For example, hyaluronic acid-based delivery systems rely on CD44-mediated endocytosis to achieve more targeting to tumor cells, while chitosan-based carriers usually possess pH-sensitive properties favoring drug delivery in the acidic tumor microenvironment (Marinho et al., 2021). Fucoidan and alginate have been well embedded in the pH-, redox-, and enzyme-stimuli-responsive systems, thus increasing their potential as components in future applications of controlled drug delivery (van Weelden et al., 2019; Haggag et al., 2023).

Polysaccharide-based systems should be considered enabling technology rather than a drug. Several preclinical studies pointed to increased pharmacokinetic profiles and reduced systemic toxicity; however, their clinical implementation is hampered by the issues of their scalable production, batch-to-batch reproducibility, limited long-term safety data, and regulatory standardization. Accordingly, the great majority of polysaccharide-based nanoplatforms are still at the proof-of-concept or early translational stages.

### 4.3 Clinical development and translational challenges

While several polysaccharide-based agents, such as lentinan and PSK, have been clinically utilized as adjuvants in cancer therapy for years, many novel or engineered polysaccharide delivery systems, as well as polysaccharides employed as biological response modifiers, are still primarily in the preclinical or early clinical development stages. Very few polysaccharides have so far achieved registration or clinical application only as immunomodulatory or supportive agents, and none are prescribed as direct antitumor drugs. Lentinan, Polysaccharide-K (PSK), and polysaccharide preparations of Ganoderma sinense have been approved in some countries as adjuvant treatments to protect against or treat the immunosuppressive effects of chemotherapy or radiotherapy and the resultant hematological toxicity to improve treatment tolerance and enhance the patient’s quality of life (Habtemariam, 2020).

Several reasons have limited its broad clinical use. The complex nature of polysaccharides-including their biological source, mode of extraction and purification, and even using chemical modifications-applies to quality control and consistency, complicating issues at the batch-to-batch level. Many are also poorly understood concerning their pharmacokinetics through biodistribution and metabolism, which further complicates rational dosage optimization and biomarker-based evaluation (Yu et al., 2018; Ye et al., 2023; Chen et al., 2021). From a regulatory standpoint, these products are more complex than most small molecules in many respects; hence, a separate framework must be established based on compositional fingerprinting and functional and biological characterization.

Most clinical evidence stems from small, heterogeneous, or region-specific studies advocating supportive benefits rather than conclusions regarding antitumor efficacy. Large, well-conceived, sufficiently powered clinical trials are indeed needed to prove the safety, efficacy, and positive role polysaccharides can play as one element among many in today’s multimodal cancer therapy.

### 4.4 Translational outlook and implications for precision oncology

Polysaccharides are unlikely to replace existing approved modalities; instead, they offer the potential to enhance these treatments through their complex modulation of biological signaling networks. The future development of polysaccharides will depend on standardized production quality, in-depth structure-function relationship studies, and scientifically driven clinical trial designs incorporating relevant biomarkers and pharmacodynamic endpoints. Systems biology and network pharmacology approaches will help delineate specific polysaccharides with clear benefits for particular therapeutic conditions (Hopkins, 2008; Yang et al., 2023; Yang et al., 2025).

Concurrently, advances in biomaterials engineering and nanotechnology are significantly expanding the potential of polysaccharides as multifunctional delivery platforms. In the context of precision oncology, this entails tailoring polysaccharide-based interventions to specific tumor molecular profiles, immunophenotypes, or microenvironmental features. For instance, surface functionalization and inherent receptor affinity enable subtype-adapted targeting—such as the use of hyaluronic acid for CD44-overexpressing triple-negative breast cancer models—thereby supporting precise, context-specific delivery strategies. In parallel, stimulus-responsive polysaccharide platforms facilitate broader immunotherapy, including modulation of immune checkpoints and the immunosuppressive tumor microenvironment. These diverse applications can be effectively integrated into a cohesive translational framework, provided that the structural complexity of polysaccharides is validated through rigorous analytical experimentation and balanced interpretation. (Li Q. et al., 2023; Mitchell et al., 2021; Mohanto et al., 2025; Zeng et al., 2021).

## 5 Conclusions, limitations, and future directions

Studies imply that polysaccharides possess immunomodulatory properties, exhibit antioxidant activities, help maintain redox balance, modulate inflammatory signaling pathways, and alter molecular interactions between malignant cells and their microenvironment, thereby fulfilling several of the hallmarks of cancer. In this respect, polysaccharides would be better conceived as biological response modifiers rather than cytotoxic agents in their ability to reshape dysfunctional cellular and microenvironmental networks and improve the efficacy of currently available treatments.

Some general limitations, which are the ultimate obstacles to their clinical use, are related to the polysaccharides’ anticancer potential. Structural heterogeneity in origin, isolation, purification, and chemical modification leads to poor reproducibility of polysaccharides in a specific way for mechanism studies and quality control. Most polysaccharides are yet to be adequately studied for their pharmacokinetics, biodistributions, or metabolites, which then put limits on their rational dose optimization and mechanism-based clinical trial design when established (Ferreira et al., 2015; Chen et al., 2024). However, the complexity of polysaccharide-receptor interactions, as well as the subsequent signaling, the complexity of polysaccharide-receptor interactions, as well as the subsequent signaling, also complicates an exact mechanistic explanation, especially when the results are primarily association-based, with a lack of receptor validation.

Another major limiting factor concerns evidence in clinical reality: all polysaccharides currently used clinically are supportive or adjuvants to standard therapies rather than effective anticancer treatments. Therefore, although these uses have shown good safety and an immunomodulatory effect, robust evidence regarding their antitumor efficacy and clinical benefits has yet to be provided through large, well-conducted clinical studies (Habtemariam, 2020). Moreover, current regulatory pathways, primarily developed for small molecules and conventional biologics, may not be fully optimized for structurally complex polysaccharides, necessitating adapted assessment approaches concerning functional and biological characterizations linked to structural ones.

In this light, polysaccharide preparation and characterization must reach a higher level of standardization to ensure reproducible and comparable results across studies. The integration of analytical chemistry with systems biology and network pharmacology provides a powerful framework to explore complex structure-activity relationships and system-level mechanisms, aligning with the paradigm-shifting concepts proposed by Hopkins (2008). These go hand in hand with clinical trials involving biomarkers and mechanisms that single out the best-responding patients to polysaccharide-based treatments, especially when combined with chemotherapy, radiotherapy, or immunotherapy.

In integrative cancer therapy, polysaccharides are being explored as multifunctional agents in immune modulation, therapeutic adjuvants, and drug-delivery systems. However, to realize this potential, future research must prioritize rigorous experimental design, a clear mechanistic understanding, and realistic translational expectations. Such a multidisciplinary approach will enhance the value of polysaccharide studies, ultimately contributing to the development of safer, more effective, and personalized treatment options for cancer.
